# Current Status of the X + C_2_H_6_ [X ≡ H, F(^2^P), Cl(^2^P), O(^3^P), OH] Hydrogen Abstraction Reactions: A Theoretical Review

**DOI:** 10.3390/molecules27123773

**Published:** 2022-06-11

**Authors:** Joaquin Espinosa-Garcia, Cipriano Rangel, Jose C. Corchado

**Affiliations:** Departamento de Química Física and Instituto de Computación Científica Avanzada, Universidad de Extremadura, 06071 Badajoz, Spain; ciprira@unex.es (C.R.); corchado@unex.ex (J.C.C.)

**Keywords:** theory/experiment comparison, potential energy surface, kinetics results, dynamics results, bimolecular hydrogen abstraction reactions of medium size, X + C_2_H_6_ → HX + C_2_H_5_, X ≡ H, F(^2^P), Cl(^2^P), O(^3^P) and OH

## Abstract

This paper is a detailed review of the chemistry of medium-size reactive systems using the following hydrogen abstraction reactions with ethane, X + C_2_H_6_ → HX + C_2_H_5_; X ≡ H, F(^2^P), Cl(^2^P), O(^3^P) and OH, and focusing attention mainly on the theoretical developments. These bimolecular reactions range from exothermic to endothermic systems and from barrierless to high classical barriers of activation. Thus, the topography of the reactive systems changes from reaction to reaction with the presence or not of stabilized intermediate complexes in the entrance and exit channels. The review begins with some reflections on the inherent problems in the theory/experiment comparison. When one compares kinetics or dynamics theoretical results with experimental measures, one is testing both the potential energy surface describing the nuclei motion and the kinetics or dynamics method used. Discrepancies in the comparison may be due to inaccuracies of the surface, limitations of the kinetics or dynamics methods, and experimental uncertainties that also cannot be ruled out. The paper continues with a detailed review of some bimolecular reactions with ethane, beginning with the reactions with hydrogen atoms. The reactions with halogens present a challenge owing to the presence of stabilized intermediate complexes in the entrance and exit channels and the influence of the spin-orbit states on reactivity. Reactions with O(^3^P) atoms lead to three surfaces, which is an additional difficulty in the theoretical study. Finally, the reactions with the hydroxyl radical correspond to a reactive system with ten atoms and twenty-four degrees of freedom. Throughout this review, different strategies in the development of analytical potential energy surfaces describing these bimolecular reactions have been critically analyzed, showing their advantages and limitations. These surfaces are fitted to a large number of ab initio calculations, and we found that a huge number of calculations leads to accurate surfaces, but this information does not guarantee that the kinetics and dynamics results match the experimental measurements.

## 1. Introduction

Chronologically, the understanding of chemical reactivity began with the first theoretical studies on atom–diatom reactions: potential energy surface (PES) developments and kinetics and dynamics studies. This began with the H + H_2_ paradigmatic reaction, with three electrons at play. Despite its apparent simplicity, its study represented an enormous effort, with the developments of Eyring and Polanyi in 1931 [1] based on previous studies by London [2] and completed by Sato in 1955 [3], which produced the well-known London–Eyring–PolanyiºSato (LEPS) surfaces and the first dynamics studies. The existence of a PES is a consequence of the Born–Oppenheimer approximation, i.e., the separation of nuclear and electronic motions. The PES is defined as the electronic energy (including the nuclear repulsion) of a given adiabatic electronic state at a fixed geometry and, therefore, it represents the effective potential energy for nuclear motion when the system is in that electronic state. Most thermally activated chemical reactions, the focus of the present work, involve only one PES, namely the lowest-energy one. Since then, many atom–diatom reactions have been studied based on this type of surface and other fitting strategies, and many efforts have been made to obtain more accurate PESs, describing the nuclei motion. There is a wide consensus that the field of small reactions, typically atom–diatom, is mature, although today, it is still a field of research where PESs continue to develop and be more and more accurate. See, for instance, in recent years, surfaces on H + NaH [4], K + H_2_ [5]; Li + HF [6], or even in 2021, the Si(^1^D) + H_2_ surface [7], and where the simplicity of these reactions was questioned [8], with an interesting study with the suggestive title: Is the simplest chemical reaction really so simple?

For these atom–diatom reactions, many kinetics and dynamics studies were performed, both experimental and theoretical, and the theory/experiment comparison served as a severe test of many theories. In the kinetics field, the transition-state theory or the variational transition-state theory arose as useful theories to evaluate rate constants and kinetic isotope effects. In the dynamics field, an interesting issue was which motion (vibration or translation) is more effective in driving the reaction. In 1972, Polanyi [9] proposed a series of rules which associate this problem with the location of the transition state as the reaction evolves. So, in the case of “early” transition states (they appear soon on the reaction coordinate, associated with exothermic reactions) translational energy is more effective than vibrational energy in raising reaction efficiency, while “late” transition states (they appear late on the reaction coordinate, associated with endothermic reactions) show the opposite behaviour. Associated with the first developments in the atom–diatom reactions and to understand their reactivity, the first dynamics studies, quasi-classical and quantum mechanical on the H + H_2_ reaction, appeared [10,11,12].

Current knowledge of chemical reactivity is well established in the studies on atom–diatom reactions, which is today a mature field.

After examining atom–diatom reactions, the next qualitative jump was the study of small size reactions, in which the H + CH_4_ hydrogen abstraction reaction was paradigmatic, and eleven electrons were involved. There is a great deal of literature on this reaction, which has been reviewed [13]. The important issues are that this reaction plan is related to the number of degrees of freedom, 3n − 6 = 12, as compared to 3 for atom–diatom reactions, which are related to the increase of computational cost to accurately describe the reactive system. Considering m configurations for each degree of freedom, m^3n−6^ calculations would be needed. A typical value of m is 10, and so for the H + CH_4_ system, 10^12^ energy calculations would be needed, as compared to 10^3^ in an atom–diatom reaction. This implies a great computational effort if chemical accuracy (1 kcal mol^−1^) is desirable, and it is practically unaffordable if spectroscopic accuracy (1 cm^−1^) is the aim. In addition to the previously noted issue about the role of translation or vibration on reactivity, other important issues emerged, such as bond and mode selectivity. The experimental studies were pioneered mainly by two groups, Zare’s lab and Crim’s lab [14,15,16,17,18,19,20].

Several reviews have reported advances in recent years in these small-size systems. Zhang, Clary, et al. [21] focused on recent advances in quantum scattering calculations, analyzing reactions from four atoms, for example, OH + H_2_; five atoms, as H + NH_3_; six atoms, as the prototype H + CH_4_, seven atoms, example OH + CH_4_, and reactions beyond six atoms, as H + CH_3_NH_2_. Guo and Liu [22] analyzed the control of chemical reactivity by transition state and beyond, focusing on the X + H_2_O/CH_4_, with X ≡ H, F(^2^P); Cl(^2^P), and O(^3^P), bimolecular reactions, emphasizing the developments in both experimental techniques and theoretical tools. In 2000 and 2013, Nyman and Yu [23,24] reviewed quantum approaches for bimolecular polyatomic reactions, emphasizing that rigorous quantum mechanical dynamics calculations become expensive as the size of the molecular system grows, and so justifying the use of reduced dimensionality calculations. In 2007, Truhlar and coworkers [13] presented an exhaustive review of theoretical developments of the H + CH_4_ reaction and isotope variants, focusing attention on the quest for an accurate potential energy surface for these systems, which stems from the pioneering work by Gorin in 1939 [25] using a three-electron valence bond model, up to the year 2007 (date of publication of review). Bowman, Czako, and Fu [26] analyzed the progress of high-dimensional ab initio potential energy surfaces for dynamics calculations in unimolecular and bimolecular reactions. These reviews follow the line of development in atom–diatom reactions, i.e., that theoretical knowledge of the chemical reaction is based on the accurate description of a full-dimensional potential energy surface (PES) describing the complete reactive system and the development of adequate kinetics and dynamics theories. These theories must include quantum effects, such as zero-point energy, resonances, or tunneling, which are complicated issues, especially when the dimension of the polyatomic system increases. The comparison of the theoretical results with the experimental evidence, where the development of sophisticated laser techniques, high resolution crossed molecular beam measures, or velocity map imaging experiments permit state-to-state fine descriptions of the reactive system, was a stringent test of their quality. Obviously, one has to be aware that in this comparison, the quality of the PES and the accuracy of the theoretical theories (kinetics and dynamics) are being tested. Usually, many theoretical studies note that theory/experiment discrepancies are due to the low quality of the PES, and although it is the weakest part, it is not always the case (see below).

In sum, the studies on small size systems, both experimental and theoretical, confirmed knowledge of chemical reactivity obtained with atom–diatom systems, and opened up new fields of research.

The natural jump in this development was to the analysis of medium-size reactions, which is the main aim of the present review, in which the construction of the PES for the H + C_2_H_6_ reaction by Truhlar et al. in 2006 [27] represented a pioneering work. In these last 15 years, many advances have been performed in this field, and will be reviewed below, with special attention to the theoretical plan, and focusing on hydrogen abstraction reactions on the ethane molecule,

X + C_2_H_6_ → HX + C_2_H_5_; X ≡ H, F(^2^P), Cl(^2^P), O(^3^P) and OH. (1)

In sum, based on the acquired knowledge in the studies on atom–diatom, diatom–diatom and small size polyatomic reactions, the key elements in the theoretical study of chemical reactivity are the construction of an accurate PES describing nuclear motion and the development of kinetics and dynamics methods (classical, semi-classical or quantum mechanics). These key elements are also present in the study of medium-size polyatomic reactions, although there are more problems due to the larger number of electrons and degrees of freedom. The present review aims to analyze the developments in the different reactions shown in Equation (1). However, before beginning with this analysis, we reviewed some recent findings with a special emphasis on the theoretical/experimental comparison of results, using the H + CH_4_ and F(^2^P) + NH_3_ reactions as examples.

## 2. Theory/Experiment Comparison

The comparison between theoretical results and experimental measurements represents stimulating scientific challenges, and this collaboration goes hand in hand with scientific advancement. On many occasions, the experiments represent a severe test of the quality of the theory, though sometimes the opposite occurs, and the theoretical results suggest experiments. This process has been a constant in the history of Physical Chemistry. However, one must be careful in this comparison because several factors are being analyzed and this is not always noted in the literature: firstly, sometimes experimental and theoretical measurements do not refer exactly to the same physical quantities; secondly, there are uncertainties in the experimental measures, and thirdly, when the experiment is used as a target, the theoretical tools are tested, which include the potential energy surface (PES) and the kinetics/dynamics method.

**Figure 1:**
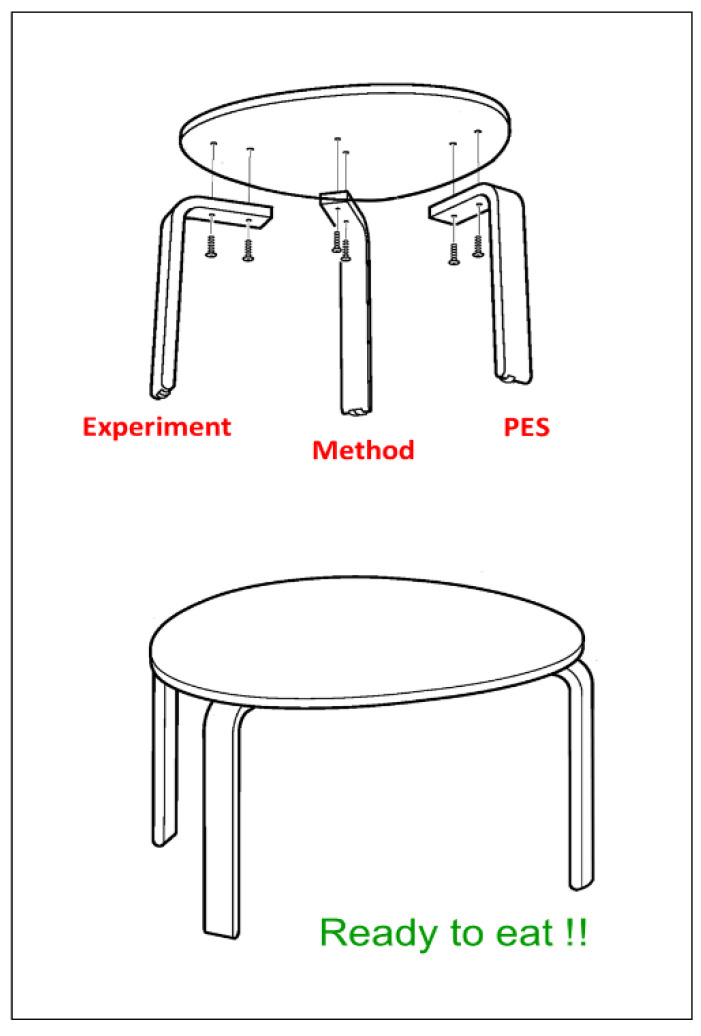
Theory/experiment comparison. The analogy of the table with three legs.

In sum, the theory/experiment comparison is a serious and complicated problem with three legs: experiment, method and PES. It is necessary to assemble the pieces in three steps to obtain a table (Figure 1). However, usually the discrepancies are blamed on the PES. Is it always the weakest leg?

Next, we present two paradigmatic cases, H + CH_4_ and F(^2^P) + NH_3_ reactions, which are small-size systems where the problems in the theory/experimental comparison are analyzed.

### 2.1. An Update of the H + CH_4_ Reaction

This reaction is a clear example of the tremendous effort in the development of full-dimensional potential energy surfaces and theoretical methods in the last two decades. Although it presents only eleven electrons, the twelve degrees of freedom make it a difficult problem. Based on high-level ab initio calculations, different strategies have been used in the construction of accurate full-dimensional PESs. Truhlar et al. [13] reviewed the advances in this field up to 2007, and we now review the research in the two last decades. Manthe et al. [28] developed ab initio-based PES using the modified Shepard interpolation method named WWM in 2004. In 2006, Zhang, Braams and Bowman [29] developed a series of surfaces, ZBBn, n = 1, 2, 3, using the permutationally invariant polynomial method (PIP), based on ~20,000 ab initio calculations. In 2009, our group [30] developed a new PES for this system using the valence bond-molecular mechanics method (VB/MM), named CBE surface, and based on ~20,000 ab initio points. Later, in 2011, Zhou et al. [31], based on ~ 30,000 ab initio points, developed the ZFWCZ surface using the modified Shepard interpolation method. Xu et al. [32] in 2014, based on ~48,000 ab initio points and using the neural network (NN) strategy, developed the XCZ surface, and finally, in 2015, Li et al. [33] increased the number of points to ~63,000 (which is an enormous computational effort) using the PIP-NN method. In all these surfaces, all ab initio calculations were performed at the CCSD(T) level or higher, using basis sets of triple-zeta quality or superior. A summary of the recent developments on the H + CH_4_ (and isotopic variants) surfaces is shown in Table 1. Note that the following high-level calculations were used: coupled-cluster with single, double, and triple excitations, CCSD(T), and explicitly correlated coupled-cluster with single, double, and triple excitations, CCSD(T)-F12, using the following basis sets of quality cc-pVTZ, VTZ, and aug-cc-pVTZ, AVTZ.

These six surfaces present barrier heights in the narrow range of 14.69–15.03 kcal mol^−1^, very close to the best estimation predicted at the CCSD(T)/aug-cc-pVQZ level, 14.87 kcal mol^−1^ [34]. Note that the six surfaces show differences of ~0.5 kcal mol^−1^ (lower than the chemical accuracy, 1 kcal mol^−1^), and they are within the best estimation with errors of ±0.2 kcal mol^−1^.

**Figure 2:**
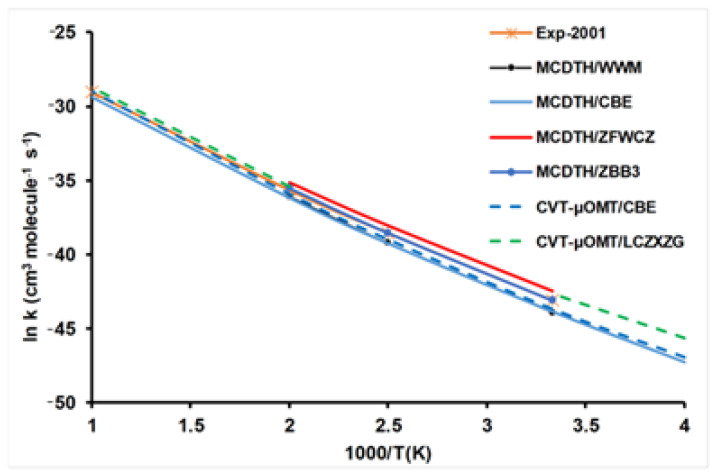
Arrhenius plots of the rate constants (cm^3^ molecule^−1^ s^−1^) for the H + CH_4_ reaction using different kinetics methods and PESs, together with experimental values for comparison. See text for acronyms.

To test the quality of these surfaces, we used kinetics (rate constants) and dynamics (excitation functions) measures because experimental data are available for comparison. First, the temperature dependence of the rate constant is analyzed (Figure 2), where the fit to experiment was computed as average predicted rates/experimental rates. To avoid possible disturbances in this comparison, theoretically, we use the same kinetics method (in this case, the multi-configuration time-dependent Hartree, MCDTH, quantum approach) on the different PESs (when they are available). With respect to the experimental measures of Sutherland et al. [35], the ZBB3 surface shows the best agreement, which practically matches the experimental data in the common temperature range of 300–500 K. The ZFWCZ surface overestimates the experimental measures by a factor <1.9, while the CBE and WWM surfaces (which agree between them) underestimate the experiment by a factor <2.0, with a better agreement at high temperatures. Figure 2 also includes kinetics results using the CVT/μOMT method (canonical variational transition-state method with tunneling of small/large curvature) based on two surfaces, CBE and the most recent LCZXZG surfaces. The rate constants are slightly underestimated and overestimated, respectively, with differences of 20–30% in the wide temperature range of 250–1000 K. In these comparisons, the kinetics method and the PES are being simultaneously tested. 

Finally, using the same PES, CBE in this case, the CVT/ μOMT rate constants give good agreement with the MCDTH quantum results, showing the error in the variational transition-state theory against quantum rate constants is small, about 20–30% in this temperature range. In this comparison, only the kinetics model is being tested. Finally, note that although the ZFWCZ surface presents the highest barrier, it shows the largest rate constants, which a priori seems counter-intuitive. This apparent controversy disappears if the tunneling effect is considered, which seems largest for the ZFWCZ surface (although, unfortunately, this data does not appear in the original paper [31]).

**Figure 3:**
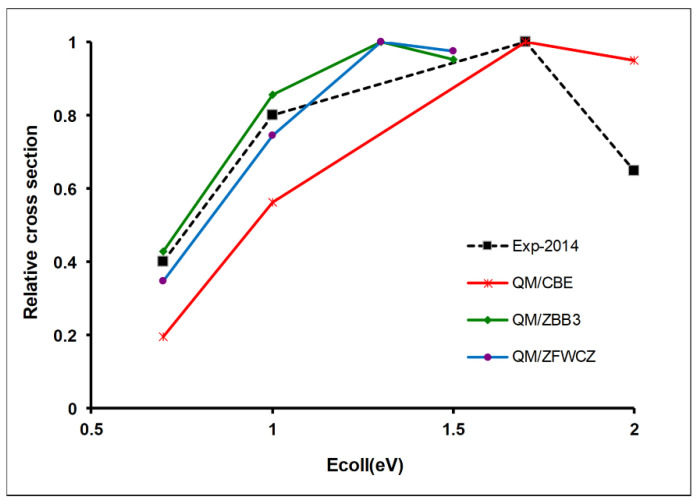
Relative integral cross-section versus collision energy (eV) for the H + CH_4_ → H_2_ + CH_3_ reaction on the ZBB3–2006 [29], CBE-2009 [30] and ZFWCZ-2011 [31] surfaces using QM methods [37]. Experimental values from Ref. [36]. For a direct comparison, each series has been scaled to its maximum value because experimental measures represent relative values.

The second test of the quality of the PESs for this reactive system was performed by using a dynamics property, the excitation function, i.e., the variation of the reaction cross-section with the collision energy. In 2011, using quantum mechanical calculations on the ZBB3, CBE, and ZFWCZ surfaces, Zhou et al. [31] performed an exhaustive analysis of the total reaction probabilities and integral cross-section, with collision energies up to 1.7 eV. Based on the error distribution theoretically obtained, they concluded that the CBE surface was less accurate than the other surfaces, especially at high energies. However, at that time, unfortunately, no experimental data were available for comparison. Later, in 2014, Pan et al. [36] reported an experimental velocity map imaging study in the energy range 0.72–1.99 eV, finding that the maximum of the excitation function was located around 1.7 eV, i.e., ~0.5 eV higher than observed with the ZBB3 and ZFWCZ surfaces. This experimental/theoretical comparison shows the inaccuracy of these two surfaces in the regime of high energies, contrary to the preliminary conclusions obtained in the absence of experimental measures. Figure 3 plots the relative integral cross-section versus collision energy (eV) for the H + CH_4_ reaction using QM calculations on the three surfaces, together with the experimental data for comparison [36].

It is worth noting that since the experimental measures represent relative values, normalized to unity, for a direct theory/experiment comparison, the results for each surface were scaled to their maximum value. It is shown that contrary to the previous conclusions, the CBE surface simulates the experimental peak at 1.7 eV better than other surfaces (ZBB3 and ZFWCZ) [37], although theory/experiment discrepancies still persist. To the best of our knowledge, similar QM calculations on the most recent LCZXZG surface have not been reported. This information should be very useful in this theory/experiment comparison. These results from the theoretical/experimental comparison lead us to an interesting conclusion: a huge amount of ab initio calculations does not guarantee in itself the accuracy of the results derived from the PES. Only the comparison with experimental results is a sound test of the quality of a theoretical method (in this case, the combination of PES and QM calculations).

Therefore, if these differences exist (both in kinetics and dynamics studies) for one of the most studied polyatomic systems, H + CH_4_, the problem will increase with the molecular size. This is the case of the reactions reviewed in the present work, where 21 or 24 degrees of freedom were involved.

### 2.2. The F(^2^P) + NH_3_ Reaction: Does a Huge Number of Points Guarantee the Accuracy of the Final Kinetics Results?

The hydrogen abstraction reaction of fluorine atoms with ammonia, F(^2^P) + NH_3_ → HF + NH_2_, is another example of a small size reaction. Contrary to the H + CH_4_ reaction, it is very exothermic, −28.14 kcal mol^−1^, and barrierless. This reaction represents a true challenge (both theoretical and experimental) because it is very fast, with possible secondary reactions, and it presents theory/experiment controversy about the temperature dependence of the rate constants. Experimentally, while Walther and Wigner in 1983 [38] reported that the rate constants were practically independent of temperature in the range 250–369 K (where large uncertainties are present), Persky [39] reported inverse temperature dependence in the range 276–327 K in 2007. Theoretically, this reaction has also been studied [40,41,42,43,44,45,46,47]. In 1997, our lab developed the first analytical potential energy surface for this reactive system, PES-1997, which was semiempirical, i.e., it was fitted to theoretical and experimental information [42]. 

**Figure 4:**
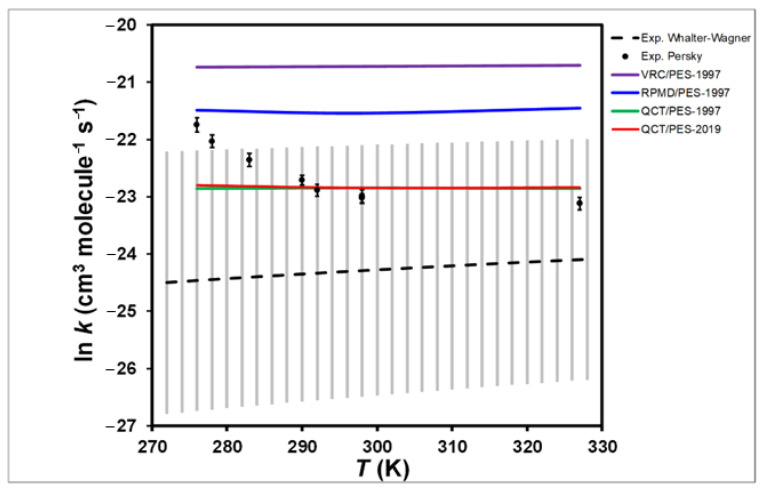
Arrhenius plots the rate constants (cm^3^ molecule^−1^ s^−1^) for the F(^2^P) + NH_3_ reaction using different kinetics methods and PESs together with experimental values for comparison. See text for acronyms.

On the PES-1997 surface, several kinetics methods were tested [46], including VRC (variational reaction coordinate), QCT (quasi-classical trajectory calculations), and RPMD (ring polymer molecular dynamics). All three methods show that the rate constants are practically independent of temperature (Figure 4). Note that an inadvertent error was found in the original paper [46]. The QCT rate constants were incorrect because they missed the factor of the electronic partition function, Equation (7) in the original paper:

(2) $$\frac{Q_{e}^{TS}}{Q_{e}^{Reactants}}=\frac{2}{4+2\exp\left(-\frac{\varepsilon }{k_{B}T}\right)}\;$$

with *ε* = 404 cm^−1^ being the spin-orbit splitting of the F(^2^P) atom. In the experimental temperature range 276–327 K, this factor is between 0.476 and 0.461. Therefore, the QCT rate constants should be multiplied by this factor, ~0.5. These corrected QCT rate constants are represented in Figure 4.

Recently, Tian et al. [47] developed an accurate PES by fitting ~41,000 ab initio points at the CCSD(T)-F12/aug-cc-pVTZ level, PES-2019, and reported kinetics results using QCT calculations. At this high level, the topography of the reaction is similar to that obtained with PES-1997, i.e., a barrierless, highly exothermic reaction, and the presence of stabilized intermediate complexes in the entrance and exit channels. With this more accurate PES, based on a huge number of points, the authors also reported that thermal rate constants have almost no temperature dependence, practically matching the previous QCT calculations on the PES-1997 surface [46]. Therefore, a huge number of points in itself does not guarantee an adequate kinetics response. Finally, in this theory/experiment comparison, experimental difficulties cannot be ruled out. Thus, the effect of very fast secondary reactions cannot be eliminated in the experiments, and the possible effect of the excited F(^2^P_1/2_) spin-orbit state was not analyzed. 

In sum, the experimental/theoretical controversy about the temperature dependence of the rate constants for this very fast reaction is an open question. This is a clear example of how we can ask ourselves if the PES is always the weakest leg.

## 3. Analysis of Medium-Size Systems

**Figure 5:**
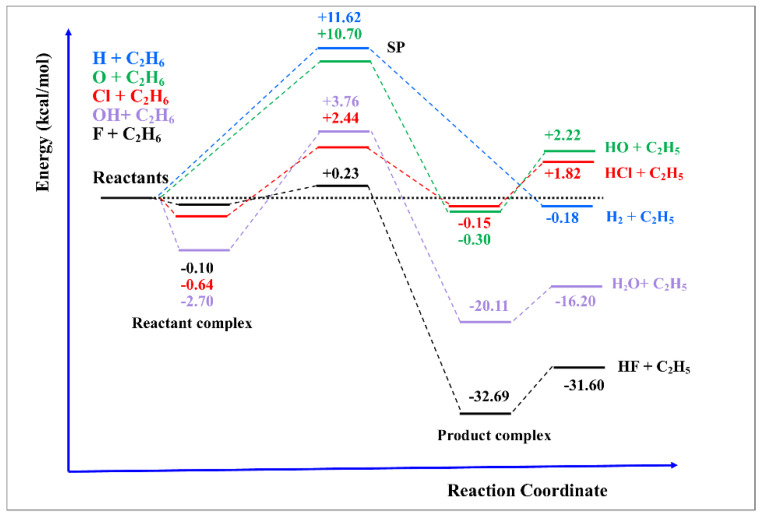
Schematic classical energy diagrams for the different bimolecular hydrogen abstraction reactions analysed in the present review. Theoretical results were obtained from our research group (see text).

Before beginning with the detailed analysis of each medium-size reaction, the main aim of this review, we now summarize the variety of topologies of these bimolecular hydrogen abstraction reactions in Figure 5, from very exothermic to endothermic reactions. In some cases, the transition state is located close to the reactants (“early”), as in the F(^2^P) + C_2_H_6_ reaction; in other cases, far from the reactants (“late”), as in the Cl(^2^P) + C_2_H_6_ reaction, and in other cases intermediate between reactants and products (“central”), as in the O(^3^P) + C_2_H_6_ reaction. In addition, the existence of intermediate complexes in the entrance and exit channels could influence the kinetics and dynamics of each reaction, and, inversely, the presence of fingerprints (as, for instance, the forward-backward behavior of the differential cross sections) in experimental measurements is evidence of the presence of these complexes. Therefore, in studying medium-size reactive systems, the older concepts already developed in atom–diatom reactions are applied.

Finally, in this review, a series of questions arose: (i) What are the similarities and differences with the reactions based on the simplest alkane, X + CH_4_?; (ii) A huge number of ab initio calculations guarantees an accurate PES, but does it also guarantee the quality of the kinetics and dynamics results?; (iii) The analyzed systems present a wide range of electrons, from 19 to 37, associated with a large computational effort. Is the PES description as accurate as in smaller systems? Are the kinetics and dynamics results in accordance with the experiment?

### 3.1. The Development of Potential Energy Surfaces

The first step in the theoretical study of any reactive system is the construction of a potential energy surface describing the nuclei motion as the reaction evolves. In the literature, many methods have been proposed, and basically, they can be classified into two broad categories: valence-bond (VB) based and molecular-orbital (MO) based surfaces, although obviously, this classification is not unique. In addition, Warshel [48] noted that it is more physical to calibrate surfaces with the first method in the study of chemical reactions, although this assertion is not universally accepted. With respect to MO-based surfaces, the last 15 years have seen spectacular advances in sophisticated methods, such as permutation invariant polynomial (PIP) [49,50,51], PIP-neural network (PIP-NN), [52] fundamental invariant-NN (FI-NN) [53,54], atomistic-NN (At-NN) [55], or kernel-based methods using permutationally invariant descriptors [56]. The different methods were developed in the original works, and so they are not revised here. Their main advantage is that they are flexible enough to pass through all the calculated points. However, (i) these methods present a high computational demand because a huge amount of high-level electronic structure calculations is necessary to describe the whole reactive system, today ~100,000 points; (ii) in addition, the points calculated for a given reactive system cannot be used for another reaction, and therefore independent sets of points need to be calculated for each particular reaction, and finally, (iii) the absence of unphysical anomalies in areas far from the sampled regions cannot be ruled out. By using MO-based surfaces, in the last four-to-five years and parallel with our research, Czako et al. [57,58,59,60,61,62,63,64] performed an impressive work analyzing reactions with ethane, X + C_2_H_6_ → HX + C_2_H_5_; X ≡ F(^2^P), Cl(^2^P), Br(^2^P), I(^2^P) and OH. These authors performed benchmark calculations at very sophisticated ab initio levels to develop full-dimensional potential energy surfaces and from them to characterize stationary points and calculate dynamics properties. These excellent results will be used in the present review to compare with the results obtained from our group using a different approach, VB-based surfaces. To the best of our knowledge, the reactions with H and O(^3^P) were not reported, nor were kinetics studies carried out by this group.

The VB-based surfaces represent an interesting alternative; they have been used from the beginning of research in this field. These methods use less ab initio information than the MO-based methods, and so the VB-based surfaces are less flexible. However, they present some advantages: (i) given the intuitive physical terms included in their development, associated with stretching, bending, or out-plane nuclei motions, which depend on a set of adjustable parameters, some of these parameters are transferable from one reactive system to another, with the consequent saving of time; (ii) given this intuitive physical character, the new PES has a certain capability to simulate zones that are not included in the fit, and (iii) when ab initio calculations of the higher level are available, the adjustable parameters can be easily refitted to fine-tune the surface.

Next, we briefly present the strategy followed by our group to develop VB-based surfaces for the title reactions. Note that a detailed term-by-term description of the PESs can be found in Ref. [65] and so is not repeated here. The analytical functional form was developed as a valence bond (VB) function augmented with molecular mechanics (MM) terms, in brief, VB/MM. In addition, a series of switching functions (developed as hyperbolic tangent functions) is included to relax the reactive system, i.e., to permit smooth changes from reactants to products while the reaction evolves. The functional form depends on a set of adjustable parameters, which were fitted to high-level ab initio calculations. As was previously noted, one of the advantages of this kind of surface is that the different terms used in its development have physical means associated with nuclei motions. So, the total potential energy, *V*, is expressed as the sum of stretching terms, *V_str_*, describing the C-H_i_ bonds, a stretching term, *V_CC_*_,_ describing the C-C bond, bending terms, *V_bending_*, out-of-plane bending terms, *V_op_*_,_ and a torsional *V_tor_* term:

*V* = *V_str_* + *V_CC_* + *V_bending_* + *V_op_* + *V_tor_* (3)

Compared with the simpler potential describing reactions with methane [30,66,67,68], X + CH_4_, Equation (3) includes the terms *V_CC_* and *V_tor_*, which were absent in the reactions with methane. The first term, *V_str_*, represents the stretching terms describing the six C-H bonds and it is developed as the sum of six London–Eyring–Polanyi (LEP) potentials. It depends on 26 adjustable parameters:

(4) $$V_{\mathrm{stret}}=\sum_{i=1}^{6}V_{3}\left(R_{{\mathrm{CH}}_{i}},R_{\mathrm{CCl}},R_{H_{i}\mathrm{Cl}}\right)$$

The second one, *V_CC_*, represents the C-C bond in ethane (which was absent in reactions with methane) and is developed by a Morse function, depending on three adjustable parameters:

(5) $$V_{CC}=D_{CC}^{1}{\left[1-\exp\left\{-\alpha_{CC}\left(R_{CC}-R_{CC}^{o}\right)\right\}\right]}^{2}$$

The *V_bending_* term is developed as the sum of harmonic potentials with respect to the reference angles in ethane, *θ^o^_ij_*, and the corresponding force constants, *k_i_*. It represents the bending motions of the reactive system and depends on 22 adjustable parameters:

(6) $$V_{bending}=\frac{1}{2}\sum_{i=1}^{5}\sum_{j=i+1}^{6}k_{ij}^{0}k_{i}k_{j}{\left(\theta_{ij}-\theta_{ij}^{0}\right)}^{2}$$

The bending out-of-plane motions of the reactive system are represented by *V_op_*, which is a quadratic-quartic potential. It is developed in function of the respective force constants *f*_Δ*i*_ and *h*_Δ*i*_, and depends on six parameters:

(7) $$V_{\mathrm{op}}=\sum_{i=1}^{6}f_{\Delta_{i}}\sum_{\begin{array}{l} j=1\\ j\neq i \end{array}}^{6}{\left(\Delta_{ij}\right)}^{2}+\sum_{i=1}^{6}h_{\Delta_{i}}\sum_{\begin{array}{l} j=1\\ j\neq i \end{array}}^{6}{\left(\Delta_{ij}\right)}^{4}$$

where the angle Δ*_ij_* measures the deviation from the reference angle $\theta_{ij}^{0}$

(8) $$\Delta_{ij}=acos\left(\frac{\left(\vec{q_{k}}-\vec{q_{j}}\right)\times \left(\vec{q_{l}}-\vec{q_{j}}\right)}{\left\|\left(\vec{q_{k}}-\vec{q_{j}}\right)\times \left(\vec{q_{l}}-\vec{q_{j}}\right)\right\|}\times \frac{{\vec{r}}_{i}}{\left\|{\vec{r}}_{i}\right\|}\right)-\theta_{ij}^{0}$$

with (*q_k_* − *q_j_*) and (*q*_l_ − *q_j_*) being two vectors between three hydrogens on each carbon and *r_i_* the vector between the carbon and each of the hydrogens directly bonded to it. The *V_op_* term permits to relax the systems from tetrahedral to planar structures in reactants and products, respectively. Finally, another motion absent in reactions with methane is the torsion motion in the C-C bond. This motion is represented by the *V_tor_* term, given by:

(9) $$V_{tor}=\overset{3}{\underset{i=1}{\sum }}\overset{3}{\underset{l=1}{\sum }}\frac{v_{3}}{3}(+\cos\gamma )\;\times T\left(w_{1},w_{2}\right)$$

where *v*_3_ is the torsional barrier height, *γ* the torsional angle and *T*(*w*_1_,*w*_2_) a switching function depending on two parameters. In total, *V_tor_* depends on three adjustable parameters. Equation (3), represents the general potential when ethane is attacked by an atom, forming ethyl radical and a diatom as products. However, when ethane is attacked by a diatom, forming ethyl radical and a triatomic product, it is necessary to include a new term. For instance, for the OH + C_2_H_6_ → H_2_O + C_2_H_5_ reaction, a new term describing the water formation, *V_H2O_*, is included (see, for instance, Ref. [69] for more details). Basically, it consists of two terms, the O-H bond in the hydroxyl free radical reactant, described by a Morse potential, and the H-O-H bending motion in the product, described by a harmonic potential. The *V_H2O_* term depends on three adjustable parameters. In sum, the analytical PESs developed in our group to describe the reactions with ethane [65,70,71,72,73], X + C_2_H_6_ → XH + C_2_H_5_; X ≡ H, F(^2^P), Cl(^2^P), O(^3^P) and OH, and reviewed in the present paper, depend on 60–63 adjustable parameters, as compared to 33–36 parameters describing the reactions with methane, which obviously represents a severe difficulty in the fitting process.

Once the functional form has been developed, the second step in the development of analytical PESs is the fitting process. At the beginning of our research with polyatomic systems [74,75] in the 1990s, and due to computational limitations, the PESs were fitted to theoretical and experimental information and therefore, they can be classified as semiempirical surfaces. Today, the situation has changed, and the fitting process is carried out using exclusively high-level ab initio calculations. For the title reactions, we used the CCSD(T)/cc-pVTZ level to optimize and characterize all stationary points (reactants, products, saddle point and reactant and product complexes if they exist) and the reaction path. Usually, the energetic description from this level is poor, and to improve it, we used explicitly-correlated calculations and larger basis sets. The influence of the correlation method and basis set can be seen in Table 2, where different methods are listed for the X + C_2_H_6_ reactions [65,70,71]. 

For the H + C_2_H_6_ reaction, for instance, taking as a reference the standard enthalpy of reaction, ΔH_r_(298K), obtained from the respective standard enthalpies of formation [76], −3.7 kcal mol^−1^, the correlated methods with large basis sets simulate this data. The saddle point description, barrier height, is a more sensible parameter. So the basis set of quality triple-zeta overestimates the barrier, larger basis sets being necessary to converge the results. The effects of correlation and basis set are more pronounced in the case of the reaction with halogen atoms [65,70], which are also listed in Table 2 due to the larger number of electrons involved. In these cases, the experimental enthalpies of reaction [76], ΔH_r_(298K) = −35.73 and −2.67 ± 0.5 kcal mol^−1^, for the F and Cl atoms, respectively, are only reached with very high levels. For the chlorine atom, for instance, the decrease of the barrier height with the level is noticeable, from 9.82 kcal mol^−1^ when the very simple MP2/6–31G(d,p) level is used, to 1.77 kcal mol^−1^ when explicitly-correlated methods are used. Note that when the spin-orbit coupling in the Cl(^2^P_3/2_,^2^P_1/2_) electronic states is considered, [76], i.e., 0.84 kcal mol^−1^, the barrier height is 2.61 kcal mol^−1^. These values simulate reasonably well the relativistic all-electron CCSDT(Q)/CBS-quality (where CBS means complete basis set) benchmark data from Czako et al. [57], with differences of about 0.5 kcal mol^−1^. For the sake of completeness, note that the geometries of all stationary points (reactants, products, and saddle point) were optimized at the MP2/6–31G(d,p) and CCSD(T)/cc-pVTZ levels, using the latter for posterior energy calculations using higher ab initio calculations: CCSD(T)/aug-cc-pVTZ, CCSD(T)/cc-pV5Z and CCSD(T)-F12/aug-cc-pVTZ levels. We observe that the CCSD(T)/cc-pVTZ level simulates better the experimental geometries of reactants and products than the lower MP2 level (see more details in the original papers, [65,70,71]). Using the CCSD(T) optimized geometries, the harmonic vibrational frequencies were calculated and used in the enthalpy corrections without scale.

**Figure 6:**
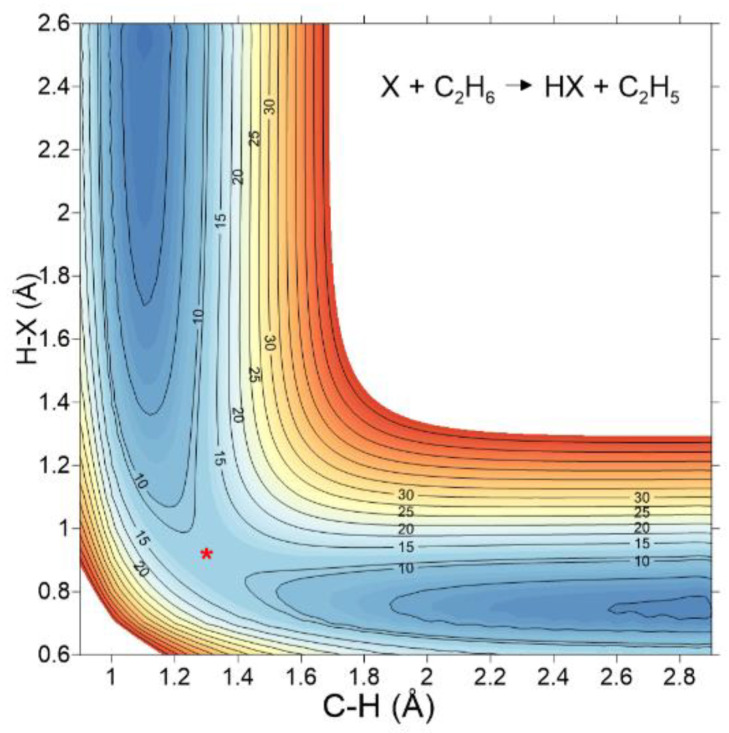
General equipotential contour plot for the X + C_2_H_6_ reactions based on analytical PESs. Reactants and products appear clearly in the plot, while the saddle point is denoted as (*). In this case, we use as a model the reaction with hydrogen atom from Ref. [71].

Using this high-level ab initio information as input data, we fitted the adjustable parameters in Equation (3) for each reaction, obtaining the corresponding analytical full-dimensional potential energy surfaces. [65,70,71,72,73]. All PESs have been deposited in our POTLIB library [77], which is freely accessible to the scientific community. Finally, note that these surfaces present many similar traits because they represent hydrogen abstraction reactions on ethane. Figure 6 shows a general two-dimensional equipotential contour plot, where the breaking C–H and forming H–X bonds are represented. These representations are useful because they show a smooth, continuous, and differentiable behaviour, where unphysical features are absent. Obviously, the original representation for each particular reaction can be found in the original papers [65,70,71,72,73].

### 3.2. The Theoretical Tools: A Compromise between Accuracy and Capacity

Always assuming an accurate global PES describing the reactive system. Obviously, the full-dimensional quantum mechanical (QM) methods are the most accurate because they describe quantum phenomena, such as zero-point energy (ZPE), tunneling, and resonances. The use of these methods in polyatomic systems has reached a significant development in the last three decades due to the increase in computer resources. Their main disadvantage is the enormous computational cost, so economical alternatives such as reduced dimensionality QM calculations or QM calculations at total angular momentum J = 0 have been implemented and applied in several polyatomic systems. However, as previously noted in point (iii) of Section 3, in these last cases, one must be careful in comparison with experiments.

An economical and widely used alternative in the study of polyatomic systems is the quasi-classical trajectory (QCT) methods [78]. However, their main disadvantage is that they are classical in nature, and so issues such as ZPE, tunneling, and resonances are not considered. In the kinetics study, different methods have been specifically proposed, such as variational transition-state theory with multidimensional semiclassical tunneling corrections (VTST/MT) [79,80], or more recently, the ring polymer molecular dynamics (RPMD) method [81,82]. Although other methods have been proposed and applied in polyatomic systems, we only consider these methods herein because they are the ones used in our group and analyzed in the present review.

Finally, and before beginning the analysis of the theoretical results, some points of caution must be taken into account, which sometimes goes unnoticed in the literature: (i) when different theoretical approaches are compared on the same PES, the theoretical approach used is tested; (ii) when theoretical methods are compared with experiments, both the PES and the theoretical approach are simultaneously tested, and (iii) sometimes theory/experiment comparison is not always made on the same footing, i.e., different physical properties are measured in each case, which is especially interesting in the analysis of dynamics properties. Finally, note that in the analysis of the theoretical results, we will go from averaged measures to state-to-state measures, i.e., from a “macroscopic” to a “microscopic” point of view, and in our research, we found that in general, the “averaged” measures, such as the thermal rate constants, are reasonably reproduced, sometimes quantitatively. However, this agreement is worse (only qualitative) when state-to-state measures are compared, such as the product vibrational distribution.

### 3.3. Theoretical Calculations Simulate Kinetics Experiments: The H + C_2_H_6_ Reaction

As noted in Figure 5, this reaction is practically thermoneutral with a high barrier, 11.62 kcal mol^−1^, which is a good candidate for tunneling effects. Therefore, the QCT method is not suitable to study, and we analyze here the VTST/MT and RPMD methods based on the PES-2019 surface [71,83], comparing the theoretical results with experiments. This reaction has been widely studied, and Baulch et al. [84] reviewed the experimental information in the temperature range 300–2000 K, recommending the following rate constant expression (cm^3^ molecule^−1^s^−1^):

k(T) = 1.23 × 10^−11^ (T/298)^1.50^ exp(−31.01/RT) (10)

**Figure 7:**
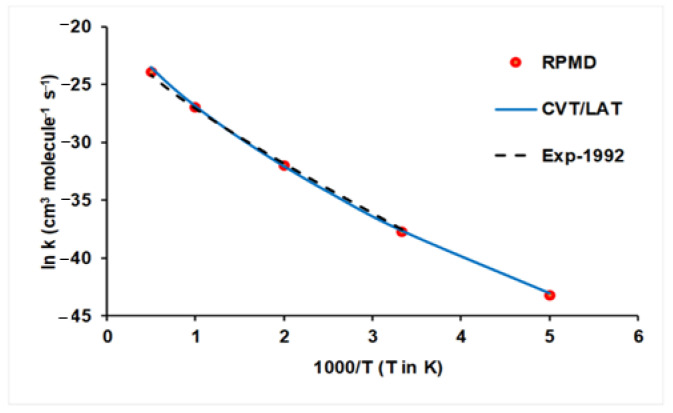
Arrhenius plots of the H + C_2_H_6_ thermal rate constants (cm^3^ molecule^−1^ s^−1^) computed using the PES-2019 surface in the temperature range 200–2000 K. Blue line: CVT/LAT rate constants; red circles: RPMD rate constants; black dashed line: experiment from Ref. [84].

Figure 7 shows the corresponding Arrhenius plots using the VTST/MT (in this case, we use the canonical version of the VTST theory and the least-action tunneling transmissions coefficient, LAT [85], in brief. CVT/LAT) and RPMD approaches together with the experimental data for comparison. The two theoretical approaches show excellent agreement between them, with differences of less than 30% in the wide temperature range (note that as both approaches are using the same PES-2019 surface, this comparison is testing the kinetics approach), and both simulate the experimental evidence (note that now both the PES and the theoretical tool are being simultaneously tested). In this particular reaction, the agreement between both approaches occurs at both low temperatures, where the tunneling contribution plays the more important role, and at high temperatures, where the recrossing effect is more important. This theory/experiment good agreement can be explained because of the high barrier, which diminishes some of the limitations of the VTST approach, such as variational or recrossing effects, and because of the absence of intermediate complexes, which make the surface fitting process easier. In addition, it shows the capacity and versatility of the PES-2019 surface to describe this reactive system. In this sense, the H + C_2_H_6_ reaction could be considered an ideal model.

**Figure 8:**
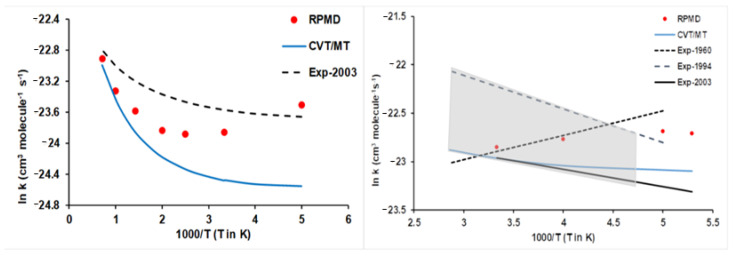
Arrhenius plots of the Cl(^2^P) + C_2_H_6_ (**left panel**) and F(^2^P) + C_2_H_6_ (**right panel**) thermal rate constants (cm^3^ molecule^−1^ s^−1^). Blue line: CVT/MT rate constants; red circles: RPMD rate constants. In the left panel, black dashed line: experiment from Ref. [86]. In the right panel, the experimental values 1960, 1994, and 2003, from Refs. [87,88,89], respectively.

However, a question quickly arises: Is the good agreement between theory/experiment a general tendency of the hydrogen abstraction reactions with ethane? The answer is pessimistic. The presence of intermediate complexes in the entrance and exit channels together with lower barrier heights, as in the case of reaction with halogens (Figure 5), makes the situation very different, clearly worsening the theory/experiment agreement (Figure 8). For the Cl(^2^P) + C_2_H_6_ reaction, the VTST/MT rate constants show large deviations from experiments [86], with differences by a factor of 2 at 200 K and smaller at high temperatures. The RPMD approach slightly improves the agreement with experiments, but over a temperature range of 200–300 K, it presents the opposite temperature dependence. The F(^2^P) + C_2_H_6_ reaction presents the largest theory/experiment discrepancies, highlighting the discrepancies between different experimental measures [87,88,89] and between theoretical approaches. Currently, it is difficult to establish a clear cause for these discrepancies, but we think in a series of problems, such as invalidity of the Born–Oppenheimer approach at low temperatures, inaccuracies of the PES and known limitations of the VTST/MT and RPMD kinetics approaches, without ruling out experimental difficulties. Obviously, with these discrepancies, one would be wary of speculating on the possible causes.

**Figure 9:**
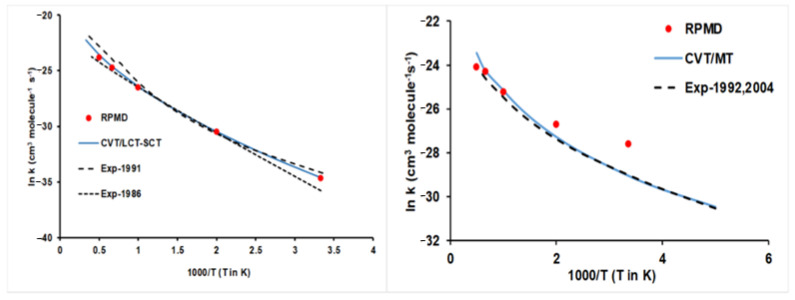
Arrhenius plots of the O(^3^P) + C_2_H_6_ (**left panel**) and OH + C_2_H_6_ (**right panel**) thermal rate constants (cm^3^ molecule^−1^ s^−1^). Blue line: CVT/MT rate constants; red circles: RPMD rate constants. In the left panel, black dashed line: experiment from Refs. [91,92]. In the right panel, experimental values from Refs. [86,93].

Finally, an intermediate situation was found for reactions with oxygen species, O(^3^P), and OH + C_2_H_6_ reactions [73,90]. Figure 9 shows the corresponding Arrhenius plots, together with the experimental evidence for comparison [86,91,92,93]. It seems that high barriers, 10.70 and 3.76 kcal mol^−1^ (Figure 5), respectively, favour a better fitting of the PESs, diminishing the errors with respect to the ab initio input data, and a better kinetic description of the reactive system, thus diminishing the errors associated with the kinetics methods.

### 3.4. The Theoretical Dynamics Description Worsens the Agreement with Experiments

Throughout our research over the years, we have found that, in general, a theoretical/experimental agreement worsens as more detailed dynamic magnitudes are compared. We found [94] that reasonable agreement occurs when the average magnitudes, such as total available energy or product energy distribution, are being compared, and a poor agreement when more “quantic” magnitudes, such as population in different product vibrational states, are compared. Obviously, many of the problems with detailed “quantum” dynamics properties will be solved when full-dimensional QM calculations are carried out on these medium-size polyatomic systems. With these limitations present, we analyze the dynamics of these reactions using QCT calculations, from “average” to state-to-state properties.

(A) The product energy partitioning between different motions: internal energy in the XH product, f_int_(XH), and in the C_2_H_5_ product, f_int_(C_2_H_5_), or as energy in translation, f_trans_, is an important average dynamics property, which permits us to know the role of the ethyl radical in the dynamics, i.e., does it act as a spectator of the reaction? Note that the internal energy corresponds to the sum of the vibrational and rotational motions in each product. The product energy partitioning for the reactions with ethane is listed in Table 3 using the QCT outcomes, where f_int_(XH), f_int_(C_2_H_5_) and f_trans_ are the fractions of the available energy appearing in the internal energy of the XH product, the internal energy of the ethyl radical and translation, respectively. Concurrently, for the exothermic F(^2^P) and OH + C_2_H_6_ reactions, the largest fraction of the available energy is deposited as product vibration, HF(v) and H_2_O(v), respectively, ~70%, in the remaining reactions, the largest fraction is deposited as translational energy, ~60%, reproducing the experimental evidence when it is available. In addition, in all the analysed reactions, the ethyl radical presents significant internal energy (between 12 and 27%), so it has a significant role in the dynamics of the reaction. In brief, it does not act as a spectator of the reaction. In general, the theoretical/experimental agreement is only qualitative, although the reaction with chlorine shows excellent agreement. These discrepancies could be due to known limitations of the QCT calculations, mainly, the ZPE violation problem, since this theory is classical, and/or to inaccuracies of the PESs, without ruling out experimental problems. For instance, in the H + C_2_H_6_ reaction, German et al. [95] experimentally used two collision energies, 1.6 and 0.7 eV, due to the fact that the reactant hydrogen atoms were yielded by photolysis of precursor HI molecule at 266 nm at two laboratory translational energies, associated with the I(^2^P_3/2_) and I(^2^P_1/2_) atoms (quantum yields of 66 and 34%, respectively).

(B) The product vibrational distribution represents the most severe test to check the quality of the theoretical tools used, the PES + dynamic method, because it is a state-to-state dynamics property that presents a strong quantum character. After each reactive trajectory, i.e., from the QCT outcomes, the final coordinates and momenta are used to calculate vibrational and rotational actions, which are rounded to the lowest integer (in units of *ħ*) to obtain the quantum states of the products. For the diatomic products, HX(v), we use the Einstein–Brillouin–Keller (EBK) semiclassical quantization of the action integral as implemented in the venus code [78]. In the case of reactions producing a diatomic product, HX(v), i.e., H, F(^2^P), and O(^3^P), Table 4 lists the product vibrational distributions, the HF(v) product being the most significant and the most studied. Note that the Cl(^2^P) reaction is not reported because it was theoretical and experimentally studied at the HCl(v = 0) vibrational state. For the HF(v) vibrational distribution the theoretical tools (PES + QCT) do not reproduce the experimental evidence, with the largest difference in the description of the HF(v = 0) ground state. While the experiments from Nesbitt et al. [96] reported a population of 16%, the theoretical results yield less than 1%. In the original paper [99], we find that due to the classical nature of the QCT calculations, an artificial energy transfer exits from the C_2_H_5_ radical to the HF product, disfavouring the population of the HF(v = 0) ground-state.

(C) Does a huge number of points for constructing the PES ensure the quality of the results of the dynamics? There are few dynamic studies in polyatomic systems comparing different high-level potential energy surfaces. The case of the F(^2^P) + C_2_H_6_ reaction, with many HF(v) vibrational states populated, presents an excellent opportunity for this comparison. Recently, two very different surfaces have been developed for this system: a VB-based surface developed in our lab [70] in 2018 and a MO-based surface developed by Czako et al. in 2020 [61]. The latter was fitted to many points obtained using very high explicitly correlated electronic structure calculations, considering large basis sets and scalar and spin-orbit relativistic corrections. In this sense, these calculations represent a benchmark for this polyatomic system, so the root means square (RMS) errors in the PES fitted are smaller than in the case of the VB-based surface [103]. However, in the analysis of the HF(v) vibrational distribution (Table 4, MO column), they found the same discrepancy with experiments as that found using the VB-based surface. So with respect to experiment of Nesbitt et al. [96], the HF(v = 0) is underestimated, while the highest vibrational states, HF(v = 2 and 3), are overestimated. In sum, although a huge number of ab initio calculations guarantees an accurate PES, it does not always guarantee the quality of the results of the dynamics.

## 4. Conclusions

The logical advances in the theoretical study of chemical reactivity began with the study of triatomic reactive systems in the middle of the 20th century. These advances continued with the study of small polyatomic systems, such as benchmark reactions with methane, to the end of the 20th century, and at the beginning of the 21st century, the study of medium-size systems, such as reactions with ethane being the natural next step in this development. In the present review, we focus attention on this last issue by analyzing several hydrogen abstraction reactions with ethane, X + C_2_H_6_ → HX + C_2_H_5_, X ≡ H, F(^2^P), Cl(^2^P), O(^3^P), and OH.

Firstly, in the theory/experimental comparison, theoretical tools such as electronic structure calculations, potential energy surface, and kinetics/dynamics approaches are being tested. However, it is usual in the literature to find that the discrepancies are blamed on the PES, and here we have shown that this is not always the case. Limitations of the kinetics and dynamics approaches are sometimes responsible for the discrepancies without ruling out experimental uncertainties. Unfortunately, this issue has gone unnoticed in the literature.

Secondly, in the last two decades, there has been a spectacular development in electronic structure calculations, reaching benchmark results, where the explicitly correlated F12 approaches [104] are today considered the state-of-the-art. These calculations were used in the development of recent PESs for polyatomic reactions. Obviously, a huge amount of these calculations guarantees the quality of the PES, but it does not always guarantee the accuracy of the kinetics and dynamics results. This problem increases when state-to-state dynamics properties are considered because they depend more on the dynamic tools used.

Thirdly, in reactions with ethane and compared with methane, what is the role of the ethyl radical in this reaction? While in the first studies, it was considered that this role was negligible, i.e., it acts as a spectator, more recent studies, both theoretical and experimental, have shown that it plays a noticeable role in the dynamics. Note that the step to the propane molecule, the following alkane in the series CH_4_ → C_2_H_6_ → C_3_H_8_, where a noticeable bending motion in the carbon chain is present, could produce more important dynamics changes.

Finally, we have found that the theory/experiment comparison worsens when we go from average, macroscopic properties to state-to-state, microscopic properties. The quantum character increases in this direction. Only full-dimensional QM calculations will solve the theory/experiment discrepancies in the latter properties.

In sum, the actual status of medium-size systems reached a degree of maturity similar to that for smaller systems as methane did ten to twenty years ago. The development in electronic structure calculations in potential energy surfaces where the Gaussian process regression (GPR) methods represent a promising alternative to huge amounts of those calculations. Furthermore and above all, the use of full-dimensional QM calculations shows significant promise in this field.

## Figures and Tables

**Table 1:** Selected recent potential energy surfaces for the H + CH_4_ reaction based on ab initio calculations.

Method PES ^a^	Ab Initio Level	Points	Barrier ^b^	Reference
Shepard interpolation	CCSD(T)/VTZ		14.93	WWM ^c^
PIP	CCSD(T)/AVTZ	~20,000	14.78	ZBB3 ^d^
VB/MM	CCSD(T)/VTZ	~20,000	15.01	CBE ^e^
Shepard interpolation	CCSD(T)/AVTZ	~30,000	15.03	ZFWCZ ^f^
NN	CCSD(T)-F12a/AVTZ	~48,000	14.69	XCZ ^g^
PIP-NN	CCSD(T)-F12a/AVTZ	~63,000	14.69	LCZXZG ^h^

^a^ PIP, permutationally invariant polynomial; VB/MM, valence bond/molecular mechanics; NN, neural network. ^b^ Classical barrier height, in kcal mol-1. ^c^ Ref. [28]; ^d^ Ref. [29]; ^e^ Ref. [30]; ^f^ Ref. [31]; ^g^ Ref. [32]; ^h^ Ref. [33].

**Table 2:** Relative energies (with respect to the energy of reactants, in kcal mol^−1^) of the stationary points at different ab initio levels for some X + C_2_H_6_ → XH + C_2_H_5_ reactions.

	Level				
	MP2 ^a^	CC/TZ ^b^	CC/augTZ ^c^	CC/5Z ^d^	CC-F12/TZ ^e^
**H + C_2_H_6_**					
Saddle point	19.43	12.35	11.89	11.97	11.83
Products	5.08	−0.36	−0.51	−0.61	−0.60
ΔH_r_(298K)	2.67	−3.36	−3.51	−3.61	−3.60
**Cl(^2^P) + C_2_H_6_**					
Saddle point	9.82	4.59	2.68	1.87	1.77
Products	9.11	4.11	3.11	1.73	1.81
ΔH_r_(298K)	4.56	−0.44	−1.44	−2.82	−2.74
**F(^2^P) + C_2_H_6_**					
Saddle point	4.24	0.19	−1.00	-	−0.93
Products	−25.01	−28.89	−31.21	-	−32.54
ΔH_r_(298K)	−28.03	−31.91	−34.23	-	−35.56

^a^ Energy and geometry optimized at the MP2/6–31G(d,p) level. ^b^ Energy and geometry optimized at the CCSD(T)/cc-pVTZ level. ^c^ Energy at the CCSD(T)/aug-cc-pVTZ level on the geometry optimized at level b. ^d^ Energy at the CCSD(T)/cc-pV5Z level on the geometry optimized at level b. ^e^ Energy at the CCSD(T)-F12/aug-cc-pVTZ level on the geometry optimized at level b.

**Table 3:** Theoretical/experimental average fraction of the available energy in products, XH + C_2_H_5_, for the X + C_2_H_6_ reactions. Values in percentage.

	H ^a^		F(^2^P) ^b^		Cl(^2^P) ^c^		O(^3^P) ^d^		OH ^e^	
	Theo	Exp	Theo	Exp	Theo	Exp	Theo	Exp	Theo	Exp
f_int_(HX)	26	18	70	70	6	2	12	-	70	51
f_int_(C_2_H_5_)	12	-	13	-	27	30	27	-	20	-
f_trans_	62	-	17	-	67	68	61	-	10	-

^a^ H + C_2_H_6_ reaction at E_coll_ = 35 kcal mol^−1^. Experimental values from Ref. [96]. ^b^ F(^2^P) + C_2_H_6_ reaction at E_coll_ = 3.2 kcal mol^−1^. Experimental values from Ref. [96]. ^c^ Cl(^2^P) + C_2_H_6_ reaction at E_coll_ = 6.7 kcal mol^−1^. Experimental values from Ref. [97]. In this case, the HCl(v = 0, j) product was compared. ^d^ O(^3^P) + C_2_H_6_ reaction at E_coll_ = 30 kcal mol^−1^. To the best of our knowledge no experimental values are available. ^e^ OH + C_2_H_6_ reaction at T = 298 K. Experimental values from Ref. [98].

**Table 4:** Theoretical/experimental diatomic, HX(v), product vibrational distribution. Values in percentage.

	H ^a^		F(^2^P) ^b^			O(^3^P) ^c^	
	Theo	Exp	Theo	Exp	MO ^d^	Theo	Exp
v = 0	81	-	0	16	2	90	-
v = 1	19	-	3	12	8	10	-
v = 2			58	43	52		
v = 3			39	28	38		

^a^ H + C_2_H_6_ reaction at E_coll_ = 35 kcal mol^−1^. Experimental values from Ref. [96] only reported that the H_2_(v) is populated in v = 0.1, mainly in the ground-state. ^b^ F(^2^P) + C_2_H_6_ reaction at E_coll_ = 3.2 kcal mol^−1^. Experimental values from Ref. [97]. ^c^ O(^3^P) + C_2_H_6_ reaction at E_coll_ = 30 kcal mol^−1^. Experimental values from Refs. [100,101,102] only suggest that the v = 0 state is the mainly populated. ^d^ Theoretical results using QCT calculations on a MO-based surface from Ref. [61].
